# The coaching on lifestyle (CooL) intervention for obesity, a study protocol for an action-oriented mixed-methods study

**DOI:** 10.1186/s12889-017-5010-4

**Published:** 2018-01-08

**Authors:** Celeste E. van Rinsum, Sanne M. P. L. Gerards, Geert M. Rutten, Ien A. M. van de Goor, Stef P. J. Kremers

**Affiliations:** 1 Department of Health Promotion, NUTRIM School of Nutrition and Translational Research in Metabolism, Maastricht University, P.O. Box 616, 6200 MD Maastricht, The Netherlands; 2 Faculty of Health and Social Studies, Institute of Health Studies, HAN University of Applied Science, P.O. Box 6960, 6503 GL Nijmegen, The Netherlands; 3 Department Tranzo, Tilburg School of Social and Behavioral Sciences, Tilburg University, P.O. Box 90153, 5000 LE Tilburg, The Netherlands

**Keywords:** Lifestyle, Coaching, Overweight, Obesity, Physical activity, Nutrition/diet, Behaviour change, Intervention, Mixed methods, Implementation

## Abstract

**Background:**

Combined lifestyle interventions (CLIs) have proved to be effective in changing and maintaining behavioural lifestyle changes and reducing overweight and obesity, in clinical and real-world settings. In this CLI, lifestyle coaches are expected to promote lifestyle changes of participants regarding physical activity and diet. In the Coaching on Lifestyle (CooL) intervention, which takes a period of 8 to 10 months, lifestyle coaches counsel adults and children aged 4 years and older (and their parents) who are obese or are overweight with an increased risk of developing cardiovascular diseases or type II diabetes. In group and individual sessions, themes such as physical activity, dietary behaviours, sleep and stress are addressed. The aim of the present study is to monitor the implementation process of the CooL intervention and to examine how the lifestyle coaches contribute to a healthier lifestyle of the participants.

**Methods:**

This action-oriented study involves monitoring the implementation process of the CooL intervention and examining the lifestyle changes achieved by participants over time, in a one-group pre-post design using mixed methods. Methods include semi-structured interviews, observations, document analysis, biomedical parameters and questionnaires.

**Discussion:**

The added value of the CooL study lies in its action-oriented approach and the use of mixed methods, including both qualitative and quantitative research methods. The long-term coaching used in the CooL intervention is expected to have beneficial effects on sustained lifestyle changes.

**Trial registration:**

NTR6208; date registered: 13–01-2017.

## Background

Half of the Dutch adult population are currently overweight or obese, as well as 12% of the children [1]. These lifestyle-related health problems have a variety of consequences, both at personal (e.g. medical problems) and at societal level (e.g. huge global economic impact) [2, 3]. Overweight is caused by an imbalance in energy balance-related behaviours, which are complex behaviours with many underlying factors [4, 5]. Combined lifestyle interventions (CLIs) seem to be suitable interventions to support persons in initiating and maintaining changes in diet and physical activity [6].

Previous randomised clinical trials (RCTs) have shown that CLIs can successfully support children [7, 8] and adults [9, 10] in changing their lifestyle behaviours and body weight. In the Netherlands, for example, the SLIM intervention was found to be effective in reducing diabetes incidence and body weight, and in improving dietary habits among adults [11]. Although evidence from relatively controlled research settings is promising, the challenge is to make these interventions suitable for “real-world” settings [12].

Recent studies have investigated the implementation of CLIs, such as the BeweegKuur [13], SLIMMER [14] and MetSLIM [15] programmes, in real-world settings in the Dutch context for patients with type II diabetes or obesity. Lifestyle Triple P [16] and COACH [17] are Dutch examples of interventions in real-world settings for children with obesity. The conclusions of these effectiveness studies were in line with those of the controlled studies, although effect sizes were generally smaller than those found in highly controlled studies. These programmes have been shown to have positive effects on physical activity and dietary behaviours, often accompanied by improved quality of life and a decrease in body mass index (BMI), waist circumference and other metabolic risk factors [13–17].

However, process evaluations of real-world CLIs have indicated implementation barriers in terms of a lack of multidisciplinary collaboration and insufficient skills among primary care professionals, as well as lack of time to optimise coaching [18, 19]. One of the major barriers for patients was the fact that their health insurance did not fully cover the costs of the intervention [18].

The identification of implementation issues, such as those reported above, has led to the design of the Coaching on Lifestyle (CooL) intervention. The lifestyle coaches in this intervention are professionals with special postgraduate training and play a key role in the intervention by coaching adults and children, who are obese or are at high risk of obesity, to help them achieve a sustained healthier lifestyle. These lifestyle coaches are thus expected to occupy a new position in the Dutch health care system [20–22], are currently in an experimental condition financed under the basic health insurance system and will function as linking pins between primary care professionals and public health professionals. In group and individual sessions, the lifestyle coaches address themes such as physical activity, dietary behaviours, sleep and stress.

The aim of the present study is to monitor the implementation of the CooL intervention and to investigate how lifestyle coaches contribute to a healthier lifestyle of the participants. This study uses an action-oriented approach, implying that results of observations are also used as input to improve the content or implementation process of the intervention. The assumption is that when opportunities and barriers are identified and adjustments are made, valid recommendations can be made for optimising the role of the lifestyle coaches in the prevention chain of chronic lifestyle-related health problems, such as obesity and overweight.

## Methods

### Design

In this action-oriented study we monitor the implementation process of the CooL intervention and the lifestyle changes achieved by participants over time, in a one-group pre-post design, using mixed methods. The process is studied by means of group and individual interviews, observations and document analysis (qualitative). The changes over time among participants are examined by means of questionnaires and biomedical parameters (quantitative). This study is expected to provide an indication of the effectiveness of this intervention in terms of the degree to which patients succeed in maintaining their changed behaviours. This study protocol has been approved by the Medical Ethics Committee of the University Hospital Maastricht and Maastricht University (reference number METC 14–5-021).

**Fig. 1:**
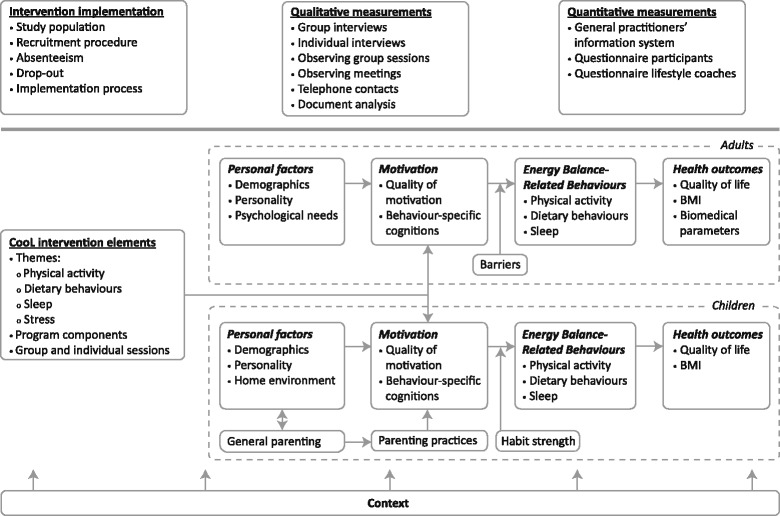
Theoretical framework for the CooL study

The theoretical framework of the study is shown in Fig. 1. The upper three boxes show the methods and measurements of this study, while the boxes within the dotted lines indicate the behavioural system of the participants, subdivided into adults and children. The energy balance-related behaviours are a combination of energy intake (i.e. dietary behaviours) and energy output (i.e. physical activity). In addition, sleeping behaviour is assumed to influence energy balance. Intervention elements are assumed to impact lifestyle changes of the participants through their influence on motivational regulation and behaviour-specific cognitions. The figure also depicts the context, which is assumed to influence the implementation process of the intervention and thus have a potential moderating influence on the changes achieved by the participants [23]. For example, when referrers are enthusiastic about the intervention, the impact of the intervention is likely to be higher.

### Study setting

The CooL study takes place in different regions within two provinces of the southern part of the Netherlands, i.e. in the province of Noord-Brabant and the south-eastern part of the province of Limburg. The CooL intervention and the data collection period for this study started in April 2014, and will continue over a period of three years.

### Study population

#### Participants

In this intervention, the lifestyle coaches counsel individuals aged 4 years and older who are obese or are at high risk of obesity (see Appendix 1). These are persons who are either obese (BMI ≥ 30) or are overweight (BMI ≥ 25) and are at increased risk of cardiovascular diseases or type II diabetes, according to the Dutch guidelines on obesity [24–26]. For children and adolescents (under 18 years), specific BMI cut-off points for the different ages are used [24]. Also, the participants must have a health insurance policy with the Dutch health insurance companies CZ or VGZ or one of their subsidiaries. Exclusion criteria are a lack of motivation to participate in the intervention and being unable to fit in a group (e.g. because of behavioural problems).

#### Lifestyle coaches

In all 13 lifestyle coaches participate in the study. All have completed a post-graduate training course at the Dutch Academy for Lifestyle and Health (AVLEG).They are members of the Professional Association of Lifestyle Coaches in the Netherlands (BLCN). A co-founder of the AVLEG has selected the lifestyle coaches, who participate in the project, based on their skills and their willingness to participate.

### Recruitment of participants

There are two pathways of recruitment: participants can either sign up for the intervention themselves or they are referred to the intervention by a health care professional. Practice nurses and general practitioners are instructed by “health care groups”, to which they are affiliated, to refer adult patients. Health care groups are organisations coordinating chronic care for a cluster of health care professionals. In addition, internal medicine specialists from hospitals are instructed by the project leader to refer potential adult participants. The recruitment of children mostly takes place via referral by Youth Health Care agencies (YHC). In addition, general practitioners, neighbourhood sports coaches, schools (such as those taking part in the “Healthy Elementary School of the Future” programme [27]), pedagogical workers and paediatricians have also been informed about the programme for children and asked to recruit potential participants. Participants that sign up by themselves, are instructed to get a referral from their health care professional.

### Sample size calculation

In a period of three years the goal is to include as much as participants as possible in order to learn optimally from the implementation process, with a maximum of 350 participants per target group (adults / children) due to financial resources. The sample size calculation is based on physical activity, since this is a primary behavioural goal in the intervention, and we used data from previous studies in similar target populations as a basis [28, 29]. For a difference of 140 min per week in physical activity (i.e. light activities (walking) and moderate-intensity to vigorous-intensity activities) with 80% power, 5% significance (two-sided), a standard deviation of 90 min per day and a dropout of 30%, a sample size of 235 adult participants is required. Potential clustering of effects (nesting of effects within the lifestyle coaches) has been corrected for by estimating the intracluster correlation (ρ) for physical activity at 0.006 (corresponding to a design effect or inflation factor of 1.23). In the effectiveness study of Lifestyle Triple P [16], it was concluded that within children a difference of 60 min per week in physical activity can be achieved with an intervention aimed at parents. Assuming a standard deviation of 30 min per day, 30% dropout and ρ of 0.006, a sample size of 135 participants is required to identify this difference with 80% power and 5% significance.

### Study procedure

The participants are informed in advance about the study using written information and personal information by the lifestyle coaches, and they are asked to give permission for the anonymous use of their biomedical data and the data gathered in the study, by means of an informed consent form. The participants receive four questionnaires to fill in: at baseline (T0); approximately 8 to 10 months after baseline, at the programme’s end (T1); 18 months after baseline (T2); and 24 months after baseline (T3). The baseline questionnaire is handed out by the health care professionals or the lifestyle coaches. The T1 questionnaire is handed out by the lifestyle coaches, and the T2 and T3 questionnaires are sent to the participants by the research team. This procedure and the referral guidelines have also been laid down in a protocol for the lifestyle coaches and referrers.

### CooL intervention

#### Programmes

Three programmes have been developed for the different age groups, based on examination of existing evidence-based programmes, theory-based literature and practice (Table 1):

1. Adults programme (18 years and older)
2. Adolescents programme, for adolescents (12 to 17 years) and their parents
3. Children’s programme, for primary school children (4 to 11 years old) and their parents

**Table 1:** Number of sessions per target group and per programme

Components	Children	Adolescents	Adults
Basic programme			
*Individual sessions*	10× at home	10× at home	4×
*Group sessions*	8× for the parents	5× for adolescents 1× for parents 2× for adolescents and parents	8×
Additional programme	–	–	10× individual sessions
Relapse prevention programme	Same number of sessions as basic intervention	Same number of sessions as basic intervention	Same number of sessions as basic intervention

In the basic programme for adults, participants are coached for 8 to 10 months (see Table 2 for an overview of the sessions and the themes). The groups consist of 4 to 12 participants. The adults attend 1 intake session and 1 outtake session (60 min each), 8 group sessions (90 min each) and 2 individual sessions (45 min each). During the intake session, the participant’s problem is mapped as well as their motivation to change their lifestyle behaviours. The children’s programme consists of 1 intake session and 1 outtake session at home with the family (60 min each), 8 group sessions with parents and a maximum of 8 individual sessions at home (in total 4 h). The focus is on providing the parents with tips for healthy parenting styles and for changing their child’s lifestyle to a healthier one. The programme for adolescents is a combination of those for the children and the adults, with a greater emphasis on peer influences. The intervention for the adolescents was added at a later stage of the implementation process, to close the gap between children and adults. As a result, our study will only address the children and adults in terms of the implementation process.

**Table 2:** Themes group sessions

Group sessions	Children	Adolescents	Adults
1	Awareness and behaviour change	Awareness and behaviour change	Awareness and behaviour change
2	Physical activity	Acting as a role model (only for parents)	Physical activity
3	Nutrition	Physical activity	Structured eating patterns
4	Setting boundaries and rewarding	Nutrition (including parents)	Sleep, relaxing, stresses
5	Acting as a role model	Snacking	Time-management
6	Sleep, relaxing, stresses	Sleep and relaxing	Pitfalls
7	Pitfalls and planning	Stresses and pitfalls	Relapse prevention
8	Self-regulation for the family	Self-regulation for the family (including parents)	Self-regulation

Regarding physical activity, the lifestyle coaches ensure contacts with the neighbourhood sports coaches or suppliers of local sports facilities. In order to improve their physical activity levels, participants are invited during the sessions to think about ways of incorporating low-intensity physical activity (and perhaps also higher intensity physical activity) in their daily lives. In addition, the lifestyle coaches can refer participants to other health care professionals, such as a dietician, general practitioner or physiotherapist, when other problems emerge.

If required, during the outtake session the lifestyle coaches assess the needs of each participant and the appropriateness to be included in the relapse prevention programme or the additional programme. The relapse prevention programme for adults comprises the same number of sessions as the basic programme. However, these sessions are spread over a period of 2 years and the content of the topics is more detailed, including more attention for the participants’ pitfalls and experiences. The additional programme consists of a maximum of 10 individual sessions, in which the personal factors are discussed in depth. Children and adolescents are only eligible for the additional programme.

#### Coordination of the intervention

The project leader, an external expert, functions as a project coordinator to start up and to implement the intervention in the different regions and as the chairman of the monthly regional project group meetings. These project groups consist of the lifestyle coaches, a coordinator from the local health care group or from the public health services (PHS), suppliers of local sports facilities and a care purchasing agent of the health insurance company. In addition to these project groups, there is a project steering group which is responsible at a higher level for the overall implementation and for long-term decisions, and meets twice a year. This project steering group includes representatives of a health care group or PHS, a municipal civil servant, a co-founder of AVLEG and the programme manager health care innovation of the health insurance company. Peer feedback meetings are organised twice a year with the lifestyle coaches and their supervisor, during which they address problems that occur in the implementation of the intervention.

### Qualitative measurements

The experiences of participants, lifestyle coaches, project team members and referrers are used to continually improve the intervention and to understand the effects of the intervention on the participants. Their opinions are collected by means of individual interviews, group interviews, observations and document analysis.

#### Group interviews

In addition to the information gained with the questionnaires, approximately one group interview is organised with the participants (i.e. the adults and parents) counselled by each lifestyle coach, immediately after the final group session, to gather more detailed information about the intervention and its barriers. These recorded interviews use a semi-structured interview protocol.

#### Individual interviews

Interviews are conducted with the different target groups: participants, lifestyle coaches, referrers, project group members and project steering group members. In addition to the group interviews with participants, more in-depth interviews are conducted with participants (*N* = 5) from one intervention group. Interviews are also conducted with the lifestyle coaches (*N* = 12) to gather information about their functioning, their role as pivots in the intervention and the network, and their opinion of the intervention. Furthermore, various referrers (*N* = 52) in each region are interviewed to explore the process of recruiting participants, the logistics of the referral process, barriers in the referral process and points for improvement. Finally, the project group members and project steering group members (N = 12) are interviewed to study the implementation process and to assess their opinions about the project group and project steering group meetings and the intervention. These interviews are audio-recorded.

#### Observing group sessions

One to two participant group sessions (with the theme sleeping and relaxation (for adults session number four and for children session number six), and the last group session) of each lifestyle coach are observed, to study the implementation of the intervention, the process of change within the groups and the development of the intervention over time. In addition, one adult group is observed during the entire programme in order to examine the intervention as a whole.

#### Observing meetings

The main researcher or a co-researcher participates in all the project group, steering group and peer feedback meetings to monitor the implementation process and the facilitating and impeding factors for this process. Notes are made of the observations.

#### Telephone contacts

Weekly telephone contacts between the project leader and the main researcher are held to keep track of the implementation process and to discuss the current situation. Furthermore, the project team members can always contact the main researcher by telephone or email. Notes of these contacts are again made by the main researcher.

#### Document analysis

All minutes from all project meetings, personal notes and e-mails are analysed to monitor the implementation process. Next to this, the lifestyle coaches keep track of absenteeism and drop-out in each intervention group. These documents also record the health care pathway, that is, participant recruitment and throughput towards suppliers of local sports facilities and other health professionals. Lastly, analysis of the intervention protocol of the lifestyle coaches and the participants’ workbooks provides detailed insights into the content of the intervention.

### Quantitative measurements

#### Biomedical parameters

Biomedical parameters of adults, i.e. objectively measured BMI, HbA1C, blood pressure and fasting glucose, will be retrieved from the general practitioners’ information system, provided by the health care group. The general practitioners assess these outcomes for patients at high risk of obesity, while the practice nurses monitor the patients with a chronic disease such as type II diabetes. The research team measures the participants’ BMI after the intervention. In addition, medication data and the number of consultations participants had with other health professionals are gathered by the health insurer. Children’s baseline BMI is measured by a YHC professional; BMI after the intervention (T1) is measured by the lifestyle coach, and BMI at T2 by the research team.

#### Questionnaire for participants

The participants receive four different questionnaires (i.e. T0, T1, T2 and T3), adapted to the three different age groups: the children (10 years and older), their parents and the adult participants (18 years and older) receive a different version of the questionnaire. See Fig. 1 for the theoretical framework, which includes the behavioural system of the participants. We used validated questionnaires where possible.

### Adults’ questionnaire

#### Demographic characteristics

The adults are asked at baseline about their personal characteristics, such as gender, date of questionnaire completion, date of birth (to calculate their age), body height and weight, country of birth, highest completed education (as an indicator of socio-economic status), living situation and occupational status. The educational level is subdivided into three categories: low (i.e. no education or only primary education), intermediate (e.g. secondary education), and high (tertiary education). The living situation is categorised into living together with someone (married or cohabiting) and living alone (divorced, unmarried or widowed).The occupational status is divided into being in work (paid work, voluntary work or self-employed) and not working (homemaker, unemployed/job seeker, retired/in early retirement, disabled/incapacitated or in education/studying). In the follow-up questionnaires participants only fill in their current weight and the date of questionnaire completion.

#### Personality

A Dutch translation of the Big Five Inventory (BFI) [30, 31] is used to measure five different personality dimensions (44 items): extraversion (8 items), agreeableness (9 items), conscientiousness (9 items), neuroticism (8 items) and openness (10 items). An example question on extraversion is: “I see myself as someone who is talkative.” These questions are answered on 5-point Likert scales ranging from totally disagree (1) to totally agree (5).

#### Psychological needs

In line with Self-Determination Theory [32] we use the Psychological Need Satisfaction in Exercise scale (PNSE) [33] to measure the perceived psychological needs regarding exercise. The 18 items (with a 5-point Likert scale from totally disagree (1) to totally agree (5)) are divided into three subscales (6 items per subscale): perceived competence, perceived autonomy and perceived relatedness. An example of perceived autonomy is “I feel free to exercise in my own way”.

#### Quality of motivation

The quality of motivation for physical activity among the adults is measured with the Behavioural Regulation in Exercise Questionnaire (BREQ-3) [34] comprising 23 questions (with a 5-point Likert scale from totally disagree (1) to totally agree (5)). The BREQ-3 consists of six subscales: amotivation (4 items), external regulation (4 items), introjected regulation (3 items), identified regulation (4 items), integrated regulation (4 items) and intrinsic regulation (4 items). An example question of introjected regulation is: “I feel guilty when I don’t exercise.”

The Regulation of Eating Behaviour Scale (REBS) [35] is used to measure motivation regarding diet and consists of 24 items, such as “Eating healthy is an integral part of my life”. The REBS has the same six subscales as the BREQ-3 (4 items per subscale): amotivation, external regulation, introjected regulation, identified regulation, integrated regulation and intrinsic motivation.

#### Behaviour-specific cognitions

At baseline, the participants are asked whether they experience social support for being physically active (13 items) and eating a healthy diet (10 items). For example: “In the last 3 months, family members/other persons, who are important to me, have participated together with me in physical activities.” Furthermore, a person’s self-efficacy is assessed by means of 4 items for both physical activity as well as diet. An example question is: “I think I will be able to be more physically active when I am tired.” Lastly, the intention to be physically active or to eat a healthy diet is assessed with 4 items per behaviour. These questions are answered on 5-point Likert scales ranging from never (1) to very often (5) or from totally disagree (1) to totally agree (5).

The adult participants are also asked at baseline about their knowledge of healthy lifestyle norms (4 items). Finally, at baseline there are questions about their previous attempts to lose weight and their reasons to participate in this intervention.

#### Barriers

The participants are asked about barriers regarding physical activity (11 items) and diet (10 items), with answering options ranging from totally disagree (1) to totally agree (5), such as “Performing physical activity is hard for me, because I feel ashamed when I’m exercising.”

#### Physical activity level

The International Physical Activity Questionnaire (IPAQ) [36] is used to measure the physical activity level of the adult participants. They are asked how many days a week and how many minutes a day they walked and engaged in moderate-intensity and vigorous-intensity activities during the past week. The numbers of days for these three activity levels is multiplied by the number of minutes. The number of minutes per week is summed to obtain a total activity score. The sedentary behaviour is assessed with 5 items in different domains (while traveling, at work, watching television, using a computer at home and at leisure), presented in minutes per day [37].

#### Dietary behaviours

The shortened Fat List [38] is used to measure dietary behaviours of the adults. They are asked about the number of days (on a scale from never to 7 days a week) they have breakfast, eat warm vegetables, salads or raw vegetables, fruits, consume fruit juices and sugar-sweetened beverages (including fruit beverages). The amount of vegetables is calculated by multiplying the number of days by the number of serving spoons a day (on a scale from 1 to 6 or more spoons). The same is done for the number of fruits (days multiplied by pieces [on a scale from 1 to 7 or more pieces]). Participants can fill in the number of slices of bread they eat each day and the type of bread (brown, whole wheat or white). They are also asked to report on how many days a week they eat different types of take-away food. Lastly, they are asked how many times a week they eat the following snacks: general snacks, peanuts or nuts, potato chips or cheese, pastries, candy, chocolates and cookies (on a scale of never to 7 times a week). These 7 questions are summed to obtain a total snacking score.

#### Quality of life

The adults’ quality of life (EQ-5D-3 L) [39] is measured with 5 questions. Each item measures a different health state and uses different answering options: mobility (from “no problem walking” to “confined to bed”), self-care (“no problems” to “unable to wash myself”), usual activities (“no problems” to “unable to perform”), pain/discomfort (none to extreme pain), and anxiety/depression (none to extremely anxious). Each question has three answering options, with lower scores meaning fewer problems.

#### BMI

The self-reported BMI is calculated from the reported height and weight. The weight status is classified into five categories according to international guidelines [40]: normal weight (18.5 ≤ BMI ≤ 24.99 kg/m^2^), overweight (BMI ≥ 25 kg/m^2^), obesity (BMI ≥ 30 kg/m^2^), severe obesity (BMI ≥ 35 kg/m^2^) and morbid obesity (BMI ≥ 40 kg/m^2^).

#### Process evaluation questions

The participants can report their experiences with the intervention as a whole in 13 questions, with a 5-point Likert scale ranging from totally disagree (1) to totally agree (5). One of the items is: “I am satisfied with the quality of the CooL intervention.” The assessment of the group sessions consists of 11 questions, with answering options ranging from totally satisfied (1) to totally dissatisfied (5). One of the questions is: “How satisfied are you with the content of the group sessions?” For the individual sessions, participants can express their appreciation in 6 questions (with options from totally satisfied (1) to totally dissatisfied (5)). One of them is: “How satisfied are you with the links between the individual sessions with the group sessions?”

Participants are also asked to answer 23 questions (with options from totally disagree (1) to totally agree (5)) about the knowledge and coaching skills of their lifestyle coach. An example of the skills question is: “My lifestyle coach helped me to draft a plan to achieve my goals.”

Finally, participants can indicate their perceived results. In 12 questions they are asked to report whether they have achieved their goals. One of these questions is: “I am now living a healthier lifestyle.” Additionally, the participants are asked 10 questions to evaluate if the goals they achieved match the intervention themes, for instance: “As a result of participating in the CooL intervention, I have made many small changes.”

### Parents’ questionnaire

#### Demographic characteristics

The children’s primary care givers are asked to report the same personal characteristics as the adult participants, for themselves, their partner (if any) and the child participating in the CooL intervention, viz. gender, date of filling in, date of birth, height, weight, country of birth, highest completed education, living situation and occupational status. Additionally, they are asked to describe their family composition and situation, the school year that the child is in and whether they use any form of child care.

#### Personality of the child

We use the questions on impulsivity from the Temperament in Middle Childhood Questionnaire (TMCQ) [41], which consists of 13 questions. The question “My child makes up its mind suddenly” can be answered on a 5-point Likert scale (with options from almost never applicable (1) to almost always applicable (5)) or choose the answer “never seen my child in this situation.”

#### General parenting

A shortened version, with 45 items, of the Comprehensive General Parenting Questionnaire (CGPQ) [42] is used to assess general parenting styles, with answering options with a 5-point Likert scale ranging from totally disagree (1) to totally agree (5).

The parental perceptions of their children’s behavioural problems regarding overweight and obesity, and the parents’ self-efficacy in dealing with these behaviours, are measured with the Lifestyle Behaviour Checklist (LBC) [43, 44]. Firstly, the parents are asked to report to what degree the child’s behaviour is a problem to them (25 items). For example, the statement “My child yells about food” is answered on a 7-point Likert scale from not at all (1) to very much (7). Secondly, for the same statement they can grade their own confidence to deal with the problem on a 10-point Likert scale from certain I cannot do it (1) to certain I can do it (10).

#### Parenting practices

To assess the parenting practices, 49 items from several validated questionnaires have been combined. Dutch translations of these questions have already been used in other studies. The 4 items on intake monitoring are based on the Child Feeding Questionnaire (CFQ) [45] and the 2 questions on monitoring activity are derived from Gubbels et al. [46]. Stimulation to be active and to eat a healthy diet is assessed by 5 items [46]. The constructs modelling healthy eating (4 items), food environment (4 items, defined as healthy food being available at home) and child control (5 items) are subscales of the Comprehensive Feeding Practices Questionnaire (CFPQ) [47]. Parental role modelling (8 items) and parental policies (5 items) for physical activity are derived from the Home Environment Survey (HES) [48]. Emotional (5 items) and instrumental feeding (4 items) are assessed using subscales of the Parental Feeding Style Questionnaire (PFSQ) [49]. Lastly, 3 items from the Covert Control scale [50] are used.

These questions use 5-point Likert scales ranging from totally disagree (1) to totally agree (5) or from never (1) to often (5). An example of emotional feeding is: “I give my child something to eat if s/he is feeling bored.”

#### Physical activity level

Parents fill in questions from the Local and National Youth Health Monitor (LNMJ) [51] to specify the physical activity level of their child. They are asked to report how many days a week and how many minutes a day their child watches television, sits behind the computer, plays outside, engages in sports at a sports club, has gym or swimming classes at school, walks or bikes to school, and walks or bikes during leisure time during a normal week.

#### Dietary behaviours

Dietary behaviours are measured with 17 questions [52]; for the children, these are filled in by their parents. In addition to the adult questionnaire, which is similar to the LNMJ, the parents fill in extra questions about different types of beverages: water, diet beverages, and energy or sports drinks.

#### Sleep

Parents are asked to report the number of hours their child sleeps on week days and during the weekend, using 2 open questions. The quality of sleep is examined by means of 4 questions, such as “During the last month, my child woke up during the night” [53].

#### Quality of life

The Impact of Weight on Quality of Life (IWQOL)-Kids [54, 55] is used to measure their children’s quality of life, using 27 items for the following scales: physical comfort (6 items), body esteem (9 items), social life (6 items), and family relations (6 items). The questions are answered on 5-point Likert scales from never (1) to always (5). An example of a statement on physical comfort is: “Because of my child’s weight, she/he avoids using the stairs as much as possible.”

#### BMI

BMI is recoded into BMI standardised for age and gender (i.e. BMI z-score) [24, 56]. Weight status will be recoded into different categories based on international cut-off values for overweight (BMI ≥ 25 kg/m^2^) and obesity (BMI ≥ 30 kg/m^2^) based on the BMI of adults aged 18 years and older [57].

The Children’s Body Image Scale (CBIS) [58] is used to identify the discrepancy between the child’s body image perception as reported by the child and by their parent. This is assessed with the question: “Which body picture is most like your own (child’s) figure?” In addition, the discrepancy between the actual BMI and the perceived BMI category is assessed. The perceived BMI category is measured with 1 question using a 5-point Likert scale from much too light (1) to much too heavy (5): “How would you describe your child’s weight?”

#### Process evaluation questions

The parents fill in the same process evaluation questions as the adults fill in. See description in the section on the questionnaire for adults.

### Children’s questionnaire

#### Home environment

Children are asked whether they have certain devices available at home, like a television (in their own room), laptop, tablet, mobile phone, or bike. Furthermore, there is a question asking whether the child has been bullied or teased.

#### Personality

We use the TMCQ [41] to measure impulsivity from the child’s point of view, with 13 questions. Questions such as “I make up my mind to do things all of a sudden” can be answered on a 5-point Likert scale (with options from almost never applicable (1) to almost always applicable (5)).

#### Quality of motivation

The children’s enjoyment of physical activity is assessed using the Physical Activity Enjoyment Scale (PACES) [59], and the motivational mechanisms of their physical activity behaviour by the Behavioural Regulation of Physical Activity in Children (BRePAC) [Bogaards L et al., unpublished observations] instrument, consisting of 16 and 18 items, respectively. Only the two contexts, in which children tend to be active, of the BRePAC are included in the questionnaire, viz. sporting outside school and playing outdoors. An example question from the PACES is: “When I am physically active I enjoy it”; one from the BRePAC is: “I participate in sports, but it is boring.” This is measured on a 5-point Likert scale from totally disagree (1) to totally agree (5). Seven items (2, 3, 5, 7, 12, 13 and 16) of the PACES are negatively formulated and they must be reversed before the total score can be calculated.

#### Behaviour-specific cognitions

Children’s self-efficacy is measured with 13 items on playing outside and 8 items on eating fruit. These questions, with a 5-point Likert scale ranging from very difficult (1) to totally not difficult (5), are based on the questionnaire of previous study [60]. A question about playing outside is: “I think it is difficult to play outside on most days after school.”

Attitude about eating fruit is measured by 7 questions. An example is: “Eating fruit is only necessary when I am sick.” The attitude questions use a 5-point Likert scale ranging from totally disagree (1) to totally agree (5).

#### Habit strength

We use a shortened version of the Self-Report Habit Index (SRHI) [61] to measure habit strength behaviour to assess the history of repetition, automaticity and expressing identity. The 3 questions (with a 5-point Likert scale from totally disagree (1) to totally agree (5)) are for the behaviours: engaging in sports, playing outside and eating fruit. One example is “Playing outside is something I do without thinking.”

#### Physical activity level

Children are asked whether they attend a sports club. They are asked what type of sports they play and how many days a week they spend on each type of sport. In addition, they are asked how they perceive their physical activity level, on a 5-point Likert scale from very low (1) to very high (5), and how active they are compared with their peers, on a scale ranging from a lot less (1) to a lot more (5).

#### Dietary behaviours

Dietary pattern is measured by how children perceive their fruit consumption, with answering options from very low (1) to very high (5), and how much fruit they eat compared with their peers, from a lot less (1) to a lot more (5).

### Questionnaire for lifestyle coaches

A questionnaire to assess competences is sent to all participating lifestyle coaches. This questionnaire includes items about their personality, work engagement and coaching competences. Personality is measured with the BFI [30]. For work engagement we use the 17-item Utrecht Work Engagement Scale (UWES) [62]. The statements have answering options on a 7-point scale from (0) never to (6) always/every day. An example is: “My job inspires me.” The competences are assessed with questions derived from the published profile of lifestyle coaches with a degree from a university of professional education [63], supplemented with questions about flexible working attitude, process monitoring, networking skills, entrepreneurship and innovative work attitude, derived from the competence assessment instrument for the Dutch universities [64].

### Analyses

The interviews, group interviews, observations and notes from the project team meetings and from the telephone meetings will be analysed with the Nvivo qualitative program. All interviews are audio-recorded and the several interview transcriptions are coded by two coders. Discrepancies between the two coders will be discussed until agreement is reached. The quantitative data will be analysed using SPSS version 21.0 on the basis of descriptives (i.e. means and frequencies), t-tests or, in case of skewed data distributions, non-parametric alternatives, and multivariate regressions. The absenteeism and drop-out will be analysed using descriptives and logistic regression to test for selectivity.

## Discussion

The purpose of this study protocol paper is to describe our study design to evaluate the implementation of the CooL intervention and to examine how the lifestyle coaches contribute to a healthier lifestyle of the participants. The added value of this study lies in the use of mixed methods (i.e. triangulation), which will increase the internal validity. This will give us information from different points of view, viz. those of the referrers, lifestyle coaches and participants. Furthermore, we combine self-reported data, observations and biomedical data. We are aware of the subjective view and potential prejudgements of the researchers, which we try to eliminate with these procedures.

Another strength of this study is its action-oriented approach, which helps us to implement and sustain the CooL intervention in the real-world setting. The observations of sessions and the interaction between the lifestyle coaches, the intermediaries and the research team enable the adjustment of the intervention programme and its implementation process during the intervention period. Furthermore, the lifestyle coaches can initiate discussions to change the programme based on their practical experiences gained during the implementation of the intervention. However, these programme changes make it more difficult to evaluate the intervention and its implementation process.

The use of one-group pre-post design makes us unable to draw definite conclusions of the causal relationship between the CooL intervention and the lifestyle changes achieved by participants over time. In contrast, the purpose of this study is primarily to answer the ‘how’ and ‘why’ questions, e.g. “How have the participants changed their lifestyle and how did they perceive the intervention to affect this?” We try to increase the external validity to combine the information from the different regions, so that we can distinguish important theoretical similarities.

The first results of the study are expected to be available at the end of 2017.

## Appendix 1 – Inclusion criteria

*Adults*

- ≥ 25 BMI < 30:
  - ○ 10-year risk of death from (cardiovascular diseases or type II diabetes) risk factors >5% or,
  - ○ Impaired fasting glucose or,
  - ○ Comorbidity:
    -  ■ Type II Diabetes
    -  ■ CVD
    -  ■ Sleep apnea
    -  ■ Osteoarthritis
- BMI ≥30

*Children*

- 4 to 10 years old:
  - ○ ≥ 25 BMI < 30 with a high risk of type II diabetes, which means having at least two risk factors:
    -  ■ 1st or 2nd degree relative with type II diabetes
    -  ■ Ethnicity (non-Western descent)
    -  ■ Signs of insulin resistance in children:
      -  ● Dlipidemia
      -  ● Low birth weight
      -  ● Acanthosis nigricans
      -  ● Polycystic ovary syndrome (signs of excessive testosterone production: irregular periods, excessive hair growth, excessive acne)
    -  ■ Hypertension
    -  ■ Gestational diabetes of the mother during pregnancy
  - ○ BMI ≥30
- 10 years or older:
  - ○ ≥ 25 BMI < 30 with a high risk of type II diabetes, as determined by blood tests.
    -  ■ Triglycerides: ≥ 1.7 mmol/L
    -  ■ HDL cholesterol: < 1.03 mmol/L for boys; < 1.29 mmol/L for girls
    -  ■ Fasting glucose: ≥ 5.6 mmol/L
  - ○ BMI ≥30
  - ○ ≥ 35 BMI > 40: referral to the lifestyle coach has to be discussed with the pediatrician.
